# The protective functions of heat shock proteins (HSPs) in combatting vibriosis: A review

**DOI:** 10.1016/j.cirep.2025.200236

**Published:** 2025-07-25

**Authors:** Yan Hanyu, Li Yihao

**Affiliations:** Key Laboratory of Northern Mariculture, Ministry of Agriculture and Rural Affairs, Dalian Ocean University, Dalian, 116023, China

**Keywords:** Heat shock proteins, Aquaculture, Vibriosis, Immune regulation

## Abstract

This paper is aimed at providing readers with a comprehensive understanding of the role played by the Heat Shock Proteins (HSPs) family in the physiology, immune response, and overall health of aquaculture animals. Due to the increase in environmental temperature brought about by climate change, aquaculture animals are encountering escalating challenges. They are profoundly affected by various biological or abiotic external stressors, particularly extreme temperatures, which might lead to cell damage and death, cause a reduction in immune function, significantly increase the risk of disease for the farmed animals, and exert adverse impacts on the growth and overall health of the farmed animals. Vibriosis is one of the principal bacterial infectious diseases in aquaculture, and its infection outcomes are affected by various environmental stressors. As one of the most prevalent components of cellular stress responses, HSPs play a crucial role in maintaining cellular protein homeostasis. This paper undertakes a review of the studies concerning HSPs (sHSPs, HSP40s, HSP60s, HSP70s, and HSP90s) in aquatic animals. HSPs not only fulfill the function of molecular chaperones but also activate numerous innate immune responses. They play a vital role in enhancing the host's adaptability to heavy metal pollution, salinity, and pH and exert significant functions in the resistance of aquaculture animals against Vibrio infections. Additionally, non-fatal heat stress and exogenous HSP-inducing compounds that enhance the production of HSPs within the organism can facilitate aquatic animals in reinforcing their immune responses to both biological and abiotic stressors. These studies centered on HSPs in aquatic animals are of utmost significance in the contemporary era that is confronted with the challenges of climate change.

## Highlights

• Summarized the symptoms of major aquaculture species infected by vibrio.
• Summarized the alternative names of the members of the heat shock protein family.
• Clarified the roles played by the members of the heat shock protein family.
• Summarized the strategies for using heat shock proteins.

## Introduction

As one of the fastest-growing modalities for obtaining animal protein globally, aquaculture can furnish sustainable and highly nutritious food, exerting a vital role in addressing global food security and coping with the challenges of malnutrition [1,2]. However, the survival and growth of aquaculture organisms are heavily influenced by environmental factors. In recent years, the emission of greenhouse gases and the consequent effects of global warming have led to rising seawater temperatures, which can accelerate the proliferation of toxic algae, resulting in harmful algal blooms and red tides that threaten marine aquaculture production [3,4]. Moreover, an increase of 1 - 2 °C in water temperature may induce severe physiological stress or even mortality in fish [5]. Additionally, ocean acidification driven by climate change poses significant challenges to marine aquaculture. It not only impairs shell formation in shellfish but also compromises immune function in fish, potentially affecting their growth and development [4,6]. As the continuous intensification of people's comprehension regarding the intimate nexus between the level of aquaculture and climate change occurs, the issue of how to mitigate and adapt to the impacts of climate change on aquaculture has garnered escalating attention.

*Vibrio* constitutes one of the most profuse bacterial groups within the marine milieu. Vibriosis engendered by diverse *Vibrio* species as pathogens constitutes a major disease afflicting both farmed and wild fish, thereby giving rise to considerable economic losses for the fishery sector [7,8]. A plethora of factors exert an influence on the infection of aquatic animals by *Vibrio*, encompassing life stages (juvenile and adult), host genetics (species and populations), co-infection with other pathogens (parasites and viruses), a weakened immune system occasioned by environmental stress, and immune priming resulting from previous exposure to *Vibrio* [9]. Furthermore, specific *Vibrio* species have the potential to induce human diseases. For example, *Vibrio vulnificus* and *Vibrio parahaemolyticus* can be consumed through raw or undercooked seafood, giving rise to sepsis, subsequent necrosis of the soft tissues in the limbs, and even human mortality [10,11]. *Vibrio cholerae* found in contaminated water and food has the potential to give rise to fatal diarrheal diseases [12]. Furthermore, *Vibrio harveyi* can also infect humans as an opportunistic pathogen via wounds that come into contact with seawater [13]. Consequently, the research on vibriosis is of paramount importance.

Heat shock proteins (HSPs), also designated as heat stress proteins, constitute a category of highly conserved biomacromolecules that are pervasively present in both prokaryotic and eukaryotic organisms [14]. HSPs represent one of the most prevailing components of cellular stress responses. HSPs can prevent apoptosis and cellular damage by inhibiting protein aggregation and exert substantial influences on cellular responses to both biological and abiotic stressors as well as the establishment of stress tolerance [15,16]. In aquaculture animals, various stress sources such as high heat, heavy metals, bacterial and viral infections, pH, and salinity can all induce an increase in the expression of HSPs. This induction enables aquatic animals to adapt to environmental challenges in a timely manner and maintain cellular balance, which is an important protective response [17,18].

In this review, we specifically concentrate on the functions of HSPs in aquatic animals when they are confronted with various stressors, particularly when exposed to *Vibrio* infections. By integrating relevant literature, the significance of HSPs in maintaining cell homeostasis and normal cell metabolism, as well as in immune regulation, was examined. Consequently, it might provide precious support for aquaculture, eventually attaining sustainable development and augmenting productivity within the perpetually changing climate.

## *Vibrio* infections in aquaculture animals

*Vibrio* typically colonizes the body surfaces and intestinal tracts of aquatic animals [19]. A multitude of *vibrio* strains that can be isolated from the gills and skin of healthy fish are regarded as non-pathogenic to fish [20], and they are also a constituent of the normal microbiota of mussels and oysters [21]. Nonetheless, when the external conditions of these aquatic animals are exposed to stress, they may transform into opportunistic pathogens, giving rise to the illness or even death of the hosts [22]. Thus, when environmental attributes such as temperature, salinity, pH, and nutrients fluctuate with the seasons or under the influence of the greenhouse effect, the prevalence of vibriosis in aquaculture animals changes correspondingly.

Among the vibrios that most frequently infect aquaculture animals are *Vibrio parahaemolyticus, Vibrio vulnificus, Vibrio harveyi, Vibrio alginolyticus, Vibrio splendidus* and *Vibrio anguillarum.* We shall enumerate and expound upon the main types of *vibrio* species that infect aquatic animals and their corresponding symptoms (Table 1).

**Table 1:** The predominant types of *Vibrio* species infecting aquatic animals and the corresponding symptoms.

Pathogenic *Vibrio* species	The group of diseased animals	Incidence symptoms
*V. parahaemolyticus*	fish	Body surface is red and swollen or its eyes are cloudy [23].
	shrimp	Red leg disease or gill rot may occur, leading to a reduction or cessation of food intake, a decline in physical activity, and some may be accompanied by symptoms of enteritis [24].
	molluscs	Abscesses or ulcers appear in the mantle, and there are white spots on the feet [25].
*V. vulnificus*	fish	Significant pathological changes are observed in the gills, including severe lesions. The liver exhibits signs of severe hemorrhage or hepatic steatosis. Additionally, there is evidence of hemorrhagic and ulcerative necrosis affecting the skin, stomach, intestines, spleen, and kidneys [26].
	shrimp	The lateral surface of the cephalothorax exhibits black stripes or deep brown localized lesions, accompanied by areas of necrosis [27].
*V. harveyi*	fish	The patient exhibited extensive ulceration of the body surface, which ultimately led to death [28].
	shrimp	Severe infection was observed in the hepatopancreas and lymphoid organs [29].
	molluscs	Hemolymphedema was observed surrounding the major circulatory system [30].
*V. alginolyticus*	fish	Sepsis, hemorrhage, cutaneous hyperpigmentation, and ulceration of the skin surface were observed [31].
	shrimp	Poor growth, anorexia, lethargy, muscle pallor, and mortality were observed [32].
	molluscs	The shells of the shellfish exhibited contraction, and some of their internal organs showed signs of necrosis [33].
*V. splendidus*	fish	Hemorrhaging was observed in the fins and subbranchial muscles, along with extensive necrosis in the liver, spleen, and kidneys [34].
*V. anguillarum*	shrimp	Result in bacterial sepsis [35].
	molluscs	Lead to large-scale mortality during the summer season [36].
*V. splendidus*	fish	Internal and external ulcers, abdominal distension, petechiae, necrotic tissue, lethargy, anorexia, extensive necrosis, erythema, arterial sheath hemorrhage, circulatory bleeding, formation of furuncles in muscle tissue, visual impairment, culminating in death [37].
	shrimp	
	molluscs	

## Heat shock proteins: types and functions

Cellular exposure to stressors such as high and low temperatures, oxidative stress, pesticides, environmental toxins, and pathogens can inflict damage on cellular proteins, giving rise to protein unfolding, aggregation, and denaturation [38]. Normal protein folding is of paramount importance for maintaining protein biological functions, and protein aggregation is cytotoxic. Hence, the disruption of protein homeostasis is associated with numerous diseases [39]. Heat shock proteins, being proteins that are widely distributed within cells of diverse species, play a vital role in maintaining normal cellular functions. They were identified in fruit flies (*Drosophilidae*) by the Italian geneticist Ferruccio Ritossa in 1962 [40]. Snoeckx et al. classified HSPs into six families, namely sHSPs, HSP40s, HSP60s, HSP70s, HSP90s, and HSP110s, based on the distinctions in the structure, function, and molecular mass of the amino acid sequences of the constituent proteins [41]. Through conducting a comprehensive review of the literature and the NCBI database, we have meticulously tabulated the members of the heat shock protein families and their aliases (Table 2) in order to facilitate the systematic collation of HSP-related materials.

**Table 2:** Classification and alternative designations of members within the heat shock protein family.

Protein family name	Protein family members	Alternate name
HSPB (sHSP)	HSPB1 (Gene ID: 3315)	CMT2F, HMN2B, HSP27, HSP28, Hsp25, HS.76067, HEL-S-102
	HSPB2 (Gene ID: 3316)	MKBP, HSP27, Hs.78846, LOH11CR1K, MGC133245
	HSPB3 (Gene ID: 8988)	HSPL27
	HSPB4 (Gene ID: 1409)	RA-crystallin, CRYAA, CRYA1, CTRCT9
	HSPB5 (Gene ID: 1410)	RB-crystallin, CRYAB, CRYA2, CTRCT16, MFM2, CMD1II, HEL-S-101
	HSPB6 (Gene ID: 126393)	HSP20, FLJ32389
	HSPB7 (Gene ID: 27129)	cvHSP
	HSPB8 (Gene ID: 26353)	H11, HMN2, CMT2L, DHMN2, E2IG1, HMN2A, HSP22
	HSPB9 (Gene ID: 94086)	FLJ27437
	HSPB10 (Gene ID: 4956)	ODF1, ODF, RT7, ODF2, ODFP, SODF
	HSPB11 (Gene ID: 424654)	C1orf41
DNAJ (HSP40)	DNAJA1 (Gene ID: 3301)	DJ-2, DjA1, HDJ2, HSDJ, HSJ-2, HSJ2, HSPF4, NEDD7, hDJ-
	DNAJA2 (Gene ID: 10294)	CPR3, DJ3, DJA2, DNAJ, DNJ3, HIRIP4, PRO3015, RDJ2
	DNAJA3 (Gene ID: 9093)	TID1, HCA57, Tid1-L;,Tid1-S, hTID-1
	DNAJA4 (Gene ID: 55466)	Dj4, Hsj4, MST104, MSTP104, PRO1472
	DNAJB1 (Gene ID: 3337)	HSPF1, Hsp40, Hdj1, RSPH16B, Sis1
	DNAJB2 (Gene ID: 3300)	HSJ1, HSPF3, CMT2T, DSMA5, HMNR5, HSJ-1
	DNAJB3 (Gene ID: 414061)	HCG3
	DNAJB4 (Gene ID: 11080)	CMYO21, CMYP21, DNAJW, DjB4, HLJ1
	DNAJB5 (Gene ID: 25822)	Hsc40
	DNAJB6 (Gene ID: 10049)	DJ4, DnaJ, HHDJ1, HSJ-2, HSJ2, LGMD1D, LGMD1E, LGMDD1, MRJ, MSJ-1
	DNAJB7 (Gene ID: 150353)	DJ5, HSC3
	DNAJB8 (Gene ID: 165721)	CT156, DJ6
	DNAJB9 (Gene ID: 4189)	ERdj4, MDG-1, MDG1, MST049, MSTP049
	DNAJB10 (Gene ID: 56812)	Hsj1, mDj8, Dnajb2, 2700059H22Rik
	DNAJB11 (Gene ID: 51726)	ABBP-2, ABBP2, DJ9, Dj-9, EDJ, ERdj3, ERj3, ERj3p, PKD6, PRO1080, UNQ537
	DNAJB12 (Gene ID: 54788)	Dj10
	DNAJB13 (Gene ID: 374407)	CILD34, TSARG5, TSARG6, RSPH16A
	DNAJB14 (Gene ID: 79982)	EGNR9427, PRO34683
HSP60	HSP60 (Gene ID: 3329)	HLD4, CPN60, GROEL, HSPD1, HSP65, SPG13, HuCHA60
HSPA (HSP70)	HSPA1A (Gene ID: 3303)	HEL-S-103, HSP70, HSP70–1, HSP70–1A, HSP70–2, HSP70.1, HSP70.2, HSP70I, HSP72, HSPA1
	HSPA1B (Gene ID: 3304)	HSP70–1, HSP70–1B, HSP70–2, HSP70.1, HSP70.2, HSP72, HSPA1, HSX70
	HSPA1L (Gene ID: 3305)	HSP70–1 L, HSP70-HOM, HSP70T, hum70t
	HSPA2 (Gene ID: 3306)	HSP70–2, HSP70–3
	HSPA3 (Gene ID: 281827)	–
	HSPA4 (Gene ID: 3308)	APG-2, HEL-S-5a, HS24/P52, HSPH2, RY, hsp70, hsp70RY
	HSPA5 (Gene ID: 3309)	BIP, GRP78, HEL-S-89n
	HSPA6 (Gene ID: 3310)	HSP70B
	HSPA7 (Gene ID: 3311)	HSP70B
	HSPA8 (Gene ID: 3312)	HEL-33, HEL-S-72p, HSC54, HSC70, HSC71, HSP71, HSP73, HSPA10, LAP-1, LAP1, NIP71
	HSPA9 (Gene ID: 3313)	CRP40, CSA, EVPLS, GRP-75, GRP75, HEL-S-124 m, HSPA9B, MOT, MOT2, MTHSP75, PBP74, SAAN, SIDBA4
	HSPA12A (Gene ID: 259217)	–
	HSPA12B (Gene ID: 116835)	C20orf60
	HSPA13 (Gene ID: 6782)	STCH
	HSPA14 (Gene ID: 51182)	HSP70–4, HSP70L1, MSANTD7
HSP90	HSP90AA1 (Gene ID: 3320)	EL52, HSPN, LAP2, HSP86, HSPC1, HSPCA, Hsp89,Hsp90, LAP-2, HSP89A, HSP90A, HSP90N, Hsp103, HSPCAL1, HSPCAL4, HEL-S-65p
	HSP90AB1 (Gene ID: 3326)	HSP84, HSPC2, HSPCB, D6S182, HSP90B
HSPH (HSP110)	HSPH1 (Gene ID: 10808)	HSP105, HSP105A, HSP105B, NY-CO-25

Heat shock proteins can function as molecular chaperones to direct the conformation of proteins throughout the entire life cycle of proteins. They facilitate the folding of nascent proteins during ribosomal synthesis, propel the transmembrane transport of proteins, and modulate the interactions between proteins by governing conformational changes [42]. Beyond the intracellular chaperone activity, the immune functions of heat shock proteins have also been delineated. Initially, bacterial heat shock proteins (particularly HSP60s and HSP70s), being highly immunogenic molecules that activate T cells, have attracted attention in autoimmune and inflammatory diseases [43]. Eukaryotic heat shock proteins have further been demonstrated to be carriers of antigenic peptides. The HSP/peptide complexes are internalized by APCs, such as macrophages and dendritic cells (DC), via receptor-mediated endocytosis [[44], [45], [46]]. This results in the presentation of major histocompatibility complex class I (MHC I) of HSPs-related peptide antigens and the induction of cytotoxic T lymphocytes (CTL) [47]. Moreover, some heat shock proteins, functioning as endogenous danger signals, are capable of activating APCs through the involvement of TLRs [[48], [49], [50], [51], [52], [53]]. Since the identical signalling mechanism operates in the recognition of pathogen-associated molecular patterns (PAMPs), the overexpression of heat shock proteins (HSPs) during the bacterial stimulation process is triggered by PAMP contamination, and HSPs might be implicated in pathogen recognition through binding to bacterial PAMPs [[54], [55], [56]].

Furthermore, diverse abiotic environmental stress factors, such as environmental stress (thermal stress), heavy metal toxicity, salinity, and pH, are capable of inducing the expression of HSPs in cells [57]. A considerable amount of research indicates that HSPs constitute the first line of defense against the detrimental effects of heat stress on organisms [58,59]. Under heat stress, the induction of HSP not only serves to maintain intracellular protein homeostasis but also functions to suppress cell apoptosis, thereby essentially protecting cells from stress [60]. Heavy metals present in the environment, such as Cu, Cd, Pb, and Zn, among others, may exert ecological impacts on organisms [[61], [62], [63]]. The elevated mRNA expression of HSPs assumes a crucial role in the organisms' response to heavy metal toxicity [64]. The addition of Cd to the feed is capable of inducing an increment in the expression of *Hsp90* in grasshoppers (*Oxya chinensis*) [65]. The participation of HSPs in the adaptation of fish to salinity changes has been well-documented [[66], [67], [68]]. In the Atlantic seabream (*Sparus sarba*), the expression of *HSP70* in the gills can be triggered to increase in hypo-osmotic and hyper-osmotic environments [69].

## The antivibrio role of heat shock proteins in aquatic animals

Aquaculture animals confront intricate environmental challenges throughout each stage of their growth and development, encompassing both biological and abiotic factors. Among these, diseases elicited by *Vibrio* rank among the most widespread in aquaculture, impeding the advancement of numerous countries and societies [70]. As an opportunistic pathogen, diverse biological and abiotic environmental stressors (such as temperature, oxygen, salinity, pH, malnutrition, and co-infection with other pathogens, etc.) can markedly influence the outcome of *Vibrio* infections [[71], [72], [73], [74]]. On the one hand, environmental stressors can have an impact on the immune system of the host. When the stressors are acute and short-term, the immune responses of fish enter the activation stage, thereby enhancing innate immunity. In contrast, the immune responses to chronic and long-term stressors exhibit an inhibitory effect that increases the risk of infection [75]. On the other hand, environmental stressors can concurrently affect *Vibrio* organisms [76]. Environmental stressors, including osmotic pressure, ethanol, temperature fluctuations, and iron starvation, have been demonstrated to downregulate the expression of the *Hfq* gene in *V. alginolyticus*, thereby influencing its ability to form biofilms [77]. Nonetheless, when exposed to long-term environmental stress, *Vibrio* has the capacity to maintain survival and guarantee virulence by upregulating selected genes [[78], [79], [80]]. Thus, the adaptability of aquatic animals to diverse environmental stressors, both biological and abiotic, is of utmost significance for their resistance to *Vibrio* infections.

HSPs, functioning as the principal stress proteins, play a pivotal role in the stress responses induced by diverse biological and abiotic factors. During heat stress, HSPs are expressed copiously and, through binding to heat-denatured proteins, aid in their refolding to restore normal structure and function, prevent protein aggregation and precipitation, thereby safeguarding cells from heat-induced damage. Under oxidative stress circumstances, HSPs contribute to maintaining the stability and activity of intracellular antioxidant enzymes, facilitate the repair or degradation of damaged proteins, and alleviate the detrimental impacts of oxidative damage on cells. In the event of chemical stress, for example, when exposed to toxic chemicals, HSPs can assist intracellular proteins in resisting the assault of chemicals, reducing protein misfolding and functional loss. In hypoxic stress, HSPs may be implicated in regulating cellular energy metabolism and oxygen utilization, facilitating cells' adaptation to hypoxic environments and sustaining their essential functions.

In conclusion, HSPs safeguard cells in diverse stress types through multiple mechanisms and pathways in order to accommodate various adverse environmental conditions, thereby enhancing the resistance of aquatic animals to pathogenic *Vibrio* infections. We will elaborate on the HSPs that mainly exert immune regulatory functions in aquatic animals one after another, including sHSPs, HSP40s, HSP60s, HSP70s and HSP90s, to enhance the understanding of their involvement in the immune defense mechanism of organisms.

### Small molecular heat shock proteins (sHSPs)

Small molecular heat shock proteins (sHSPs or HSPB) are low-molecular-weight chaperone proteins, whose molecular weights typically range from 15 to 30 kDa [81,82]. A characteristic of sHSPs is the existence of a conserved crystallin domain, flanked by variable N- and C-termini. Both the N- and C-termini and part of the crystallin domain are involved in substrate binding. Current research reveals that sHSPs can serve as molecular chaperones by binding to denatured proteins in an ATP-independent manner, primarily undertaking various cellular housekeeping processes, such as the folding of newly synthesized polypeptides, the refolding of metastable proteins, the assembly of protein complexes, the degradation of misfolded proteins, and the dissociation of protein aggregates [16,83]. Furthermore, sHSPs possess multiple biological roles in cell growth, differentiation, membrane fluidity, cell proliferation, cell apoptosis, homeostasis, and growth and development processes [[84], [85], [86], [87], [88]].

In *Sinonovacula constricta*, sHSP (ScsHSP) is potentially implicated in mediating heavy metal stress, and its expression level in hemocytes undergoes a significant increase concomitant with the augmentation of the amplitude of PbCl2 [89]. In *Carassius auratus*, the response of *HSP20* (*gfhsp20*) to heat shock or heat-sensitive regulatory mechanisms varies among the brain and other organs. *gfhsp20* mRNA is prominently induced by heat shock in the liver and spleen, while its expression is diminished in the brain. Additionally, *gfhsp20* mRNA also assumes a crucial role in oxidative stress and is induced in the brain, liver, and spleen under H2O2 exposure. In *Macrobrachium rosenbergii, Haliotis discus*, and *Epinephelus coioides*, upon being confronted with biotic stressors, such as bacterial or viral infections, the expression of *sHSP* mRNA undergoes a significant increase, functioning in the immune response of the organisms [[90], [91], [92]].

### The heat shock protein 40 family (HSP40s)

HSP40 proteins, also denominated as J proteins or DnaJ proteins, exhibit a characteristic J domain structure [93]. This constitutes a molecular chaperone competent in facilitating ATP hydrolysis for the regulation of HSP70. All eukaryotic cells encompass DNAJ proteins. The DnaJ protein family comprises three subtypes (DNAJA, DNAJB, and DNAJC); the structure of the DNAJA protein consists of a J domain located at the N-terminus, succeeded by a G/F domain (a domain rich in glycine/phenylalanine), a zinc finger domain, and an SBD domain (a substrate-binding domain) at the C-terminus; the structure of the DNAJB protein contains merely one J domain and a G/F domain; and the DNAJC protein solely possesses one J domain [94,95]. The interaction with HSP70 to stimulate ATPase activity and act in conjunction with HSP70 as a chaperone enables HSP40 to assist HSP70 in the processes of protein folding, assembly, and disassembly of protein complexes.

The research carried out by Jie-Li Cai et al. demonstrated that in *Amphilophus citrinellus*, it was detectable in all the examined tissues, with a predominant distribution in the cytoplasm. During the lipopolysaccharide (LPS) attack, the expression of Hsp40 was upregulated, inhibiting the suppression of nuclear factor κB (NF-κB), enhancing the activity of protein 1 (AP-1), reducing the ratio of *Bax/Bcl-2* mRNA expression, and playing a crucial role in the antibacterial immune response [96]. The *EcHsp40* in *Epinephelus coioides* was conspicuously induced by Singapore grouper iridovirus (SGIV), facilitating SGIV-induced apoptosis and exerting a significant antiviral role [97]. The *HSP40* (*PtHSP40-I*) of *Portunus trituberculatus* is capable of binding to lipopolysaccharide (LPS) and peptidoglycan (PGN), facilitating the translocation of the transcription factor dorsal from the cytoplasm to the nucleus of the hemocyte and participating in the regulation of the expression of anti-lipopolysaccharide factor (ALF) and shell proteins [98].

### The heat shock protein 60 family (HSP60s)

The research regarding HSP60s in aquatic animals is comparatively scarce when contrasted with that of other HSP families; however, it possesses recognized functions in protein folding. In response to environmental stress, the augmentation of unfolded proteins can trigger the induction of HSP60 [99]. The Gly-Gly-Met repeat motif constitutes a crucial element of CsCPN60, playing a specific role in facilitating the rearrangement of certain folding intermediates by forming a hydrophobic interface and maintaining the stability of proteins under heat stress [100]. Additionally, it functions as an antigen peptide and activate the adaptive immune response of the body [18].

The HSP60 within *Megalobrama amblycephala* is capable of participating in the innate immune response against Aeromonas hydrophila infection and exhibits sensitivity to heat stress, undergoing rapid and significant upregulation upon stimulation. Nevertheless, in *Takifugu rubripes, TrHSP60* demonstrates a relatively sluggish response to high-temperature stress, commencing a predominant increase after 48 h of heat stimulation [101]. Research suggests that HSP60 can be used as a vaccine component to stimulate protective humoral immunity. For instance, it has been verified that when the HSP60 protein is intraperitoneally injected into *Salmo salar*, it can elicit a humoral response and also confer protection upon fish under attack by Rickettsia [102].

### The heat shock protein 70 family (HSP70s)

HSP70s constitute the most abundant protein family among HSPs and have been recognized as molecular chaperones mediating protein biogenesis for a considerable period of time [103]. In both eukaryotic and prokaryotic cells, HSP70 serves as a central hub in the protein homeostasis network. Hsp70s are capable of binding and releasing unfolded polypeptide chains in an ATP-dependent manner within cells, thereby preventing incorrect molecular folding in unfolded polypeptide chains and exerting a significant role in regulating peptide folding, degradation, as well as the interactions between membranes and proteins [[103], [104], [105], [106]]. In the presence of some nucleotide exchange factors, HSP70 can collaborate with molecular chaperones possessing J domains or specific sequence motifs (His-Pro-Asp) to promote ATP hydrolysis and facilitate the correct folding of newly synthesized proteins, thereby maintaining the protein homeostasis of the host cell [107].

The activity of HSP70 within the cell nucleus, organelles, and cytosol underlies its multiple functionalities, and holds paramount significance for the long-term adaptation of aquatic animals to high-temperature environments [108]. The expression of 6 °C heat-shock *HSP70* in the liver of *Oncorhynchus mykiss* undergoes a significant increase [109]. Some studies on Antarctic fish have demonstrated that the expression of HSP70 in the liver is strikingly higher than that in the gills subsequent to heat shock [110], which constitutes a protective mechanism generated by fish to prevent organ damage in high-temperature environments [111]. In *Ctenopharyngodon idella*, HSP70 can be involved in enhancing ROS-induced autophagy and strengthening the antiviral effect against grass carp reovirus (GCRV) [112]. HSP70 in *Tegillarca granosa* stabilizes the lysosomal membrane and shields cells from the impacts of various stressors [113]. During the infection of *V. parahaemolyticus*, the knockdown of *HSP70* (*LvHSP70*) in *Litopenaeus vannamei* through RNA interference (RNAi) led to a significant reduction in the expression of multiple immune-related genes, encompassing antimicrobial peptides, cytokines, and components of the prophenoloxidase (ProPO) system. Moreover, 16S rDNA sequencing disclosed a marked increase in the relative abundance of *Vibrio* within the intestinal tract of *LvHSP70*-deficient shrimp. Additionally, decreased shrimp activity, loss of feed intake, hepatopancreatic damage, and individual deaths were witnessed, underlining the vital role of *LvHSP70* in maintaining shrimp homeostasis, regulating immune responses, and governing the intestinal microbiota [114]. Furthermore, Hsp70 also plays a crucial role in protecting the late-stage larvae of *Litopenaeus vannamei* from the influences of low pH and salinity [115]. Under the attacks of low temperature and hypoxia, the expression of *HSP70* in *Ruditapes philippinarum* is significantly upregulated [116].

### The heat shock protein 90 family (HSP90s)

Heat shock proteins with molecular weights ranging between 80 and 83 (HSP80–HSP83) pertain to the HSP90 family. Heat shock protein 90 (Hsp90), an extremely conserved ATP-dependent molecular chaperone, is implicated in protein homeostasis [117]. It constitutes an essential protein in eukaryotes and is recognized to play a role in the remodelling of hundreds of client proteins and to partake in numerous cellular functions, such as protein trafficking, signal transduction, and receptor maturation [118,119]. Hsp90 is involved in protecting organisms against environmental stressors, such as heat and cold stress, heavy metal stress, and stress caused by high or low pH values, among others. Moreover, the expression level of *Hsp90* transcripts is regarded as a significant indicator for biomonitoring of environmental toxins and stress [120]. Emerging studies also indicate that the Hsp90/Cdc37 complex is implicated in a series of cellular processes, such as cell cycle regulation, DNA and protein synthesis, and signal transductio [121].

During exposure to cadmium (Cd), the PmHsp90/PmCdc37 complex in *Penaeus monodon* enhanced the activity of the SOD enzyme, thereby reducing the MDA content in the hemolymph of the shrimp and safeguarding the shrimp against the damage caused by Cd stress[122]. In *Chlamys nobilis*, the expression level of *CnHSP90* exhibited a strong positive correlation with the total carotenoid content (TCC), suggesting that both carotenoids and HSP90 levels can enhance the heat tolerance of the noble scallop [123]. The research conducted by Ziwei Chang et al. reveals that a competition for binding to HSP90 might occur between the aryl hydrocarbon receptor (AhR) and the estrogen receptor (ER), resulting in mutual inhibition [124].

**Fig. 1:**
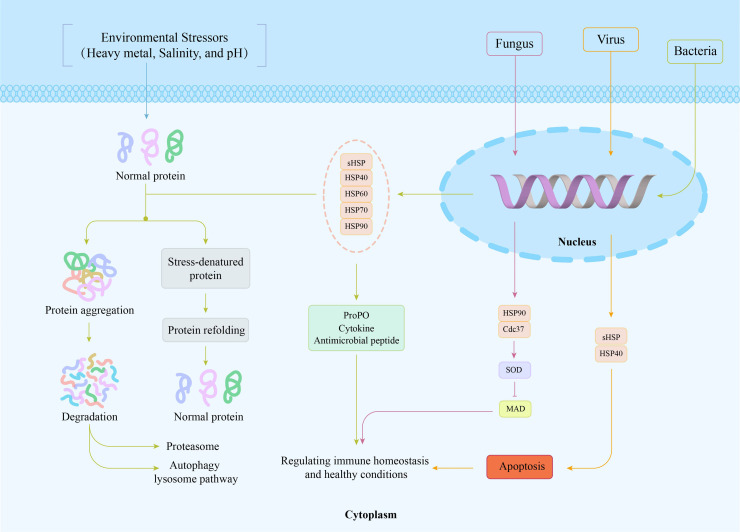
Presents the mechanism of action of intracellular heat shock proteins and a schematic illustration of their role in the immune response model of aquatic animals.

As shown in Fig. 1, under the stimulation of various biotic and abiotic stressors, misfolded proteins are produced within cells, generating cytotoxicity and threatening cell viability and homeostatic balance. Various HSP genes are induced to be expressed, restoring some of the misfolded and denatured proteins to the normal state and directly delivering protein aggregates/unfolded proteins to the ubiquitin-proteasome system or autophagy for degradation. When confronted with bacterial infection, sHSPs, HSP40s, HSP60s, HSP70s, and HSP90s are all upregulated, enhancing the activities of antimicrobial peptides and cytokines and inducing the activation of the proPO system. When encountering viral infection, the expression of sHSPs and HSP40s is upregulated, promoting cell apoptosis. Additionally, when cells are exposed to heavy metal Cd, the PmHsp90/PmCdc37 complex can increase the activity of SOD enzyme, thereby reducing MDA content and protecting cells from the damage caused by Cd stress.

## The utilization of HSPs in aquaculture health management

In the aquaculture industry, outbreaks of diseases cause significant economic losses every year (FAO, 2024). With the intensification of climate and environmental changes in recent years, the problem of aquatic animal diseases has become increasingly widespread. Currently, the conventional method of disease control in aquaculture is the use of various antibiotics. Although antibiotics are effective, they may also lead to the development of antibiotic resistance in pathogens [125]. The misuse of antibiotics can also cause bioaccumulation in aquatic products, posing a severe threat to public health and inflicting damage on the entire ecological environment [126,127]. Therefore, the greater use of HSPs in aquaculture health management can effectively avoid the environmental threats and health risks brought by antibiotics.

One of the potential applications of HSPs in aquaculture is the highly expressed HSPs induced by non-lethal short-term heat stress. Generally, the prolonged increase in the rearing environmental temperature can interfere with the normal physiological processes of aquatic animals, influencing their feed intake and vigor, reducing growth rate, weakening immunity, and even causing death in severe cases [68]. On the other hand, HSPs produced by short-term heat stress can enhance the resistance of aquatic animals to pathogenic bacteria and viruses. For instance, in a study on juvenile *P. monodon*, short-term heat stimulation increased the expression of HSP70 and reduced the replication of related viruses in the gills [128]. Another study indicated that in *Artemia franciscana*, after a 37 °C treatment for 30 min and a 6-hour recovery followed by *V. campbellii* infection, the survival rate of the heat treatment group was twice that of the untreated group.

Apart from the high expression of HSPs induced by heat stress treatment, some pharmacologically active compounds are capable of enhancing HSP synthesis. Tex-OE® is an extract of cactus (*Opuntia ficus indica*), which can boost the synthesis of HSPs in various aquatic animals [129]. It has been reported that in Artemia, Tex-OE® can induce the expression of HSP70 and upregulate two crucial immune genes of the innate immune system, namely polyphenol oxidase and cross-linking enzyme, thereby enhancing the immunity of shrimp against pathogenic *Vibrio* [130]. Because it has both nutritional and immunomodulatory functions, it can be further explored to add it on a large scale to feed to reduce antibiotic dependence.

Vaccination is capable of generating long-term protection against specific diseases and serves as an effective approach for restricting various diseases in higher vertebrates. By contrast, based on the scale and environment of aquaculture, vaccine injection is undoubtedly a challenging undertaking. Nevertheless, pathogen-derived HSP has also been employed as a vaccine, and its efficacy against bacteria and other parasites in fish has been evaluated [131,132]. The DNA vector carrying HSP70 from the parasite *Cryptocaryon irritans* has been utilized as a vaccine, and this vaccine has demonstrated enhanced protective effects against the parasite in grouper fish [132].

## Conclusion

Although HSPs act cooperatively at the cellular level, most HSP families possess specific functions exerted in distinct cellular compartments (such as the cytoplasm, endoplasmic reticulum, and mitochondria). In recent years, the unique importance of these various types of cellular functions and their specific significance in responding to particular stressors have garnered considerable attention. Nevertheless, research on aquatic animals remains inadequate. This study presents a comprehensive account of the crucial role that HSPs play in the stress adaptation and health of aquatic animals. The thermal stress responses of aquatic animals constitute a major challenge for aquaculture, influencing the growth performance, immune function, and overall health condition of aquatic animals. HSPs play a central role in alleviating the detrimental effects of external stressors by maintaining protein integrity, facilitating cell survival, and modulating immune responses. Hence, the application of non-lethal thermal stress, HSP inducers, exogenous HSP as a dietary supplement, and HSP-based vaccines might be effective strategies for the health management of aquaculture animals to enhance the stress resistance of farmed animals, and could assist in addressing the potential adverse impacts of environmental factors. In conclusion, a more profound comprehension of the regulation and function of HSPs under external stressors can provide a molecular theoretical basis for breeding stress-resistant varieties, optimizing breeding management, and achieving green and healthy breeding, guiding the development of innovative aquaculture methods to enhance the tolerance and productivity of aquatic animals when confronted with complex environmental challenges.

## CRediT authorship contribution statement

**Yan Hanyu:** Writing – original draft, Software. **Li Yihao:** Writing – review & editing, Visualization, Validation, Supervision, Resources, Project administration, Methodology, Investigation, Funding acquisition, Formal analysis, Data curation, Conceptualization.

## Declaration of competing interest

The authors declare that they have no known competing financial interests or personal relationships that could have appeared to influence the work reported in this paper.

## Data Availability

Data will be made available on request.
